# Optimising testing strategies for classification of human health and environmental hazards – A proof-of-concept study

**DOI:** 10.1016/j.toxlet.2020.10.008

**Published:** 2020-12-15

**Authors:** Emilie Da Silva, Anders Baun, Elisabet Berggren, Andrew Worth

**Affiliations:** a European Commission, Joint Research Centre (JRC), Ispra, Italy; b Department of Environmental Engineering, Technical University of Denmark, Bygningstorvet 115, 2800, Kgs. Lyngby, Denmark

**Keywords:** BPR, Biocidal Products Regulation, CLP, Classification, Labelling and Packaging, CMR, Carcinogenic, Mutagenic, toxic to Reproduction, ECHA, European Chemicals Agency, EU, European Union, GHS, Globally Harmonized System, NAMs, New Approach Methodologies, OECD, Organisation for Economic Cooperation and Development, PPR, Plant Protection Products Regulation, QSAR, Quantitative Structure-Activity Relationship, REACH, Registration, Evaluation, Authorisation and Restriction of Chemicals, STOT – RE, Specific Target Organ Toxicity – Repeated Exposure, STOT – SE, Specific Target Organ Toxicity – Single Exposure, SVHC, Substances of Very High Concern, TG, Test Guideline, vPvB, very Persistent/very Bioaccumulative, Chemical safety, Hazard identification, Risk management, CLP regulation, Alternative methods

## Abstract

This paper outlines a new concept to optimise testing strategies for improving the efficiency of chemical testing for hazard-based risk management. While chemical classification based on standard checklists of information triggers risk management measures, the link is not one-to-one. Toxicity testing may be performed with no impact on the safe use of chemicals . Each hazard class and category is not assigned a unique pictogram and for the purpose of this proof-of-concept study, the level of concern for a chemical for the population and the environment is simplistically considered to be reflected by the hazard pictograms. Using active substances in biocides and plant protection products as a dataset, three testing strategies were built with the boundary condition that an optimal approach must indicate a given level of concern while requiring less testing (strategy B), prioritising new approach methodologies (strategy C) or combining the two considerations (strategy D). The implementation of the strategies B and D reduced the number of tests performed by 6.0% and 8.8%, respectively, while strategy C relied the least on *in vivo* methods. The intentionally simplistic approach to optimised testing strategies presented here could be used beyond the assessment of biocides and plant protection products to gain efficiencies in the safety assessment of other chemical groups, saving animals and making regulatory testing more time- and cost-efficient.

## Highlights

• Testing of chemicals does not always inform their subsequent risk management.
• Optimised testing strategies can improve efficiency of classification and labelling.
• Hazard pictograms were used to reflect the protection level for a given chemical.
• Two strategies led to the same protection level and required fewer tests.
• Another strategy led to the same protection level and reduced animal testing.

## 1 Introduction

In the European Union (EU), the manufacture, import, distribution, use and disposal of chemicals is regulated by a complex interplay of more than 40 pieces of legislation (EC, 2019). However, the protection of human health and the environment is generally based on two overarching approaches, evaluating either the hazard or risk of a chemical. Under the Classification, Labelling and Packaging Regulation (CLP Regulation, (EC, 2019), there is an obligation to classify and label all chemicals according to their “intrinsic” hazards. For chemicals with certain uses (cosmetics, additives in food or feeding stuffs, plant protection products, biocidal products) or manufactured above certain tonnage levels (industrial chemicals), there is an additional requirement to conduct a risk assessment based on the intrinsic hazard and the potential exposure, and aimed at establishing safe exposure levels. Chemicals with certain hazardous properties of very high concern are banned or restricted according to the regulatory sector (*e.g.* carcinogenic, mutagenic, toxic to reproduction (CMR) or very persistent/very bioaccumulative (vPvB) substances). Although testing is not explicitly requested for the purpose of classification and labelling, it is conducted under other regulatory programmes, which often indirectly leads to risk management based on classification and labelling.

Traditionally, both the hazard-based and risk-based regulatory approaches are established on toxicity and ecotoxicity data that involve animal testing. While this historical precedent is often seen as a barrier for the uptake of non-animal methods (Sewell et al., 2017), there are in principle no legal barriers for inclusion of so-called new approach methodologies (NAMs; ECHA, 2016) not relying on animal testing (Heringa, 2014). According to the horizontal Directive 2010/63/EU on the protection of animals for scientific purposes (EC, 2010), animal testing should only be conducted as a last resort, with the ambition to eventually replace it completely. The Globally Harmonized System for Classification and Labelling of Chemicals (GHS) is updated periodically to include any relevant non-animal methods.

The CLP Regulation is based on the GHS (UN, 2019) and its purpose is to ensure a high level of protection of health and the environment, as well as the free movement of substances, mixtures and articles. According to the CLP Regulation, any hazardous substance or mixture put on the market must be classified. This is an obligation of the manufacturer or importer. The self-classification, reported to the European Chemicals Agency (ECHA) Classification & Labelling Inventory (Classification & Labelling Inventory), often leads to discrepancy in classifications of the same substance, perhaps because the data available to the different actors are not the same, the interpretation of the classification criteria has been done differently, or because the substance is on the borderline between two categories in the same class. Harmonised classification of substances is required for certain hazard classes of high concern, but there might also be a need to agree and make a legally binding harmonised classification for other reasons, *e.g.* when the substance is also subject to an authorisation scheme such as under the Biocidal Products Regulation (BPR) or the Plant Protection Products Regulation (PPR), or because there is a specific concern flagged for the use of the substance.

The more than 40 EU legal acts on the authorisation and use of substances and mixtures all refer to the classification criteria as defined in the CLP Regulation. The resulting hazard-based classifications have legal consequences on the subsequent risk management (ECHA, 2017). For example, substances classified as CMRs category 1 may be identified as substances of very high concern (SVHC) and are subject to restrictions under the regulation concerning the Registration, Evaluation, Authorisation and Restriction of Chemicals (REACH, (EC, 2006), and are severely restricted under the BPR (EU, 2012) and the PPR (EC, 2009), as well as in consumer products, for example cosmetics (EC, 2009) and toys (EC, 2009). When used in the workplace, specific safety rules apply (ILO, 1974). The extent to which these regulatory measures protect human health and the environment is difficult, perhaps even impossible, to quantify.

Specific testing strategies apply to gather, or generate, data to meet the classification criteria addressing the different hazard classes and categories. According to the current testing strategy for biocides and plant protection products, active substances must undergo toxicity evaluation for each of the hazard classes independently (EU, 2012; EC, 2009).

The classification and labelling is not only a communication tool, but also a trigger for preventive and protective actions for chemical safety. In this sense, the hazard labelling, including the pictograms, of a chemical can be regarded as a representation of the level of concern for the population and the environment.

Considering that each hazard class and category including the route of exposure is not always assigned a unique label (combination of pictograms, signal words, and standard statements) (Table 1), there is a risk for testing to be performed with no additional impact on the subsequent risk management. These overlaps and potential redundancies gave rise to the idea that an optimised testing strategy may be developed by understanding when sufficient testing has been conducted to indicate a given level of concern and when additional testing would be redundant, even if for a different endpoint.

**Table 1:** Pictograms, hazard classes, signal words, hazard statements and hazard categories from the Globally Harmonized System of Classification and Labelling of Chemicals. Table 1 shows that a single pictogram is associated with a variety of hazard classes and categories. Hazard class refers to the nature of the hazard (health or environmental endpoint). Hazard category refers to the hazard severity within a hazard class. Hazard statements are assigned to a hazard class and category to describe the nature, and, where appropriate, the degree of the hazards (UN, 2019).

Pictogram	Hazard class	Signal word	Hazard statement		Hazard category			
	Human health			1	2		3	4
GHS05: Corrosive	Skin corrosion/irritation	DANGER	Causes severe skin burns and eye damage (H314)	X				
	Serious eye damage/ eye irritation	DANGER	Causes serious eye damage (H318)	X				
GHS06: Toxic	Acute toxicity	DANGER	Fatal if swallowed (H300)	X	X			
			Toxic if swallowed (H301)				X	
			Fatal in contact with skin (H310)	X	X			
			Toxic in contact with skin (H311)				X	
			Fatal if inhaled (H330)	X	X			
			Toxic if inhaled (H331)				X	
GHS07: Harmful	Acute toxicity	WARNING	Harmful if swallowed (H302)					X
			Harmful in contact with skin (H312)					X
			Harmful if inhaled (H332)					X
	Skin corrosion/irritation	WARNING	Causes skin irritation (H315)		X			
	Skin sensitisation	WARNING	May cause an allergic skin reaction (H317)	X*				
	Serious eye damage/eye irritation	WARNING	Causes serious eye irritation (H319)		X			
	Specific target organ toxicity – single exposure	WARNING	May cause respiratory irritation (H335)				X	
			May cause drowsiness or dizziness (H336)				X	
GHS08: Health hazard	Aspiration hazard	DANGER	May be fatal if swallowed and enters airways (H304)	X				
		WARNING	May be harmful if swallowed and enters airways (H305)		X			
	Respiratory sensitisation	WARNING	May cause allergy or asthma symptoms or breathing difficulties if inhaled (H334)	X*				
	Germ cell mutagenicity	DANGER	May cause genetic defects (H340)	X				
		WARNING	Suspected of causing genetic effects (H341)		X			
	Carcinogenicity	DANGER	May cause cancer (H350)	X				
		WARNING	Suspected of causing cancer (H351)		X			
	Reproductive toxicity	DANGER	May damage fertility or the unborn child (H360)	X				
		WARNING	Suspected of damaging fertility or the unborn child (H361)		X			
	Specific target organ toxicity – single exposure	DANGER	Causes damage to organs (H370)	X				
		WARNING	May cause damage to organs (H371)		X			
	Specific target organ toxicity – repeated exposure	DANGER	Cause damage organs through prolonged or repeated exposure (H372)	X				
		WARNING	May cause damage organs through prolonged or repeated exposure (H373)		X			
	Environment			Acute	Chronic			
				1	1		2	
GHS09: Environmental hazard	Environmental hazard	WARNING	Very toxic to aquatic life (H400)	X				
			Very toxic to aquatic life with long lasting effects (H410)		X			
			Toxic to aquatic life with long lasting effects (H411)				X	

* Categories 1, 1A and 1B.

The aim of this paper is to (1) provide an overview of the hazard classes distribution of a selected, extensive dataset and describe the current testing strategy, (2) illustrate how different testing strategies can be optimised with the boundary condition of indicating the same level of concern, and (3) discuss the results obtained with regards to their applicability beyond the dataset.

This study introduces a new concept and is intended as a thought-starter for developing more efficient, but equally effective, classification and labelling of chemicals as the basis for hazard-based risk management. Ultimately, optimised testing strategies have the potential to significantly reduce the use of experimental animals, and make regulatory testing more time- and cost-efficient.

## 2 Materials and methods

### 2.1 Data acquisition and mining in the dataset

Active substances in biocidal products and plant protection products were selected as the dataset to establish new testing strategies. Under the current EU legal framework, data are required for most toxicity endpoints for their harmonised classification. Consequently, the resulting CLP classification based on complete datasets evaluated by expert panels can be considered robust. The following definitions are used: a hazard class refers to the nature of the hazard (health or environmental endpoint), a hazard category refers to the hazard severity within a hazard class, and hazard statements are assigned to a hazard class and category to describe the nature, and, where appropriate, the degree of the hazards (UN, 2019).

A retrospective analysis of hazard classification and labelling of all active substances in biocides and plant protection products in the EU was conducted. All approved biocidal active substances were retrieved from the ECHA database (Biocidal active substances database). The Classification & Labelling Inventory database (Classification & Labelling Inventory) was used to match harmonised classification and labelling data (hazard class, hazard category, and hazard statement) to each CAS registry number. The dataset included 147 active substances approved between 1 January 2009 and 1 June 2020, which are distributed over 22 product type (PT) categories. The two most frequent categories were PT08 Wood preservatives and PT18 Insecticides, acaricides and products to control other arthropods. In the case of active substances in plant protection products, data were downloaded from the EU plant protection products database (Plant protection products database). Hazard classes and hazard categories were attributed according to the registered classification in line with the CLP Regulation (EC, 2019). The dataset included 1256 active substances approved between 1 January 2002 and 20 May 2019, which are distributed over 20 product type categories (Table 1 in Supplementary Information for exclusion criteria). The three most represented groups were fungicides, insecticides and herbicides. Lastly, the biocides dataset and the plant protection products dataset were combined. The resulting dataset, comprising 1403 substances, is referred to as the “full dataset” hereafter.

Data analysis was conducted using R Studio (version 3.6.1). To map the hazard classes of the active substances in the dataset, plots were drawn using the *ggplot2* package.

### 2.2 Development of optimised testing strategies

Information on the hazards identified using the classification criteria of the CLP Regulation is communicated through specific labels (pictograms, signal words and standard statements) with the aim to protect human health and the environment (Table 1). For the purpose of this proof-of-concept study, we propose that the pictograms referring to health hazards (GHS05, GHS06, GHS07 and GHS08) and environmental hazards (GHS09) may be used to indicate the level of concern. Based on this premise, three testing strategies were built with the boundary condition that an optimal approach must indicate the same level of concern while requiring less testing (testing strategy B), prioritising NAMs (testing strategy C) or combining the two considerations (testing strategy D). The current testing strategy (*i.e.* parallel testing for all hazard classes) is named testing strategy A.

Irrespective of the testing strategy developed (*i.e.* B, C, or D), a two-step optimisation was carried out (Fig. 1 in Supplementary Information).

For the first step, the pictograms were ranked based on two conditions. Article 26 of the CLP Regulation (EC, 2019) states that the pictogram GHS07 shall never appear when GHS06 appears and it shall not be used for *skin sensitisation*, *skin corrosion/irritation* and *serious eye damage/eye irritation* when GHS05 appears, or when GHS08 appears for *respiratory sensitisation*. In addition, some hazard classes can trigger the pictogram GHS07 or another pictogram depending on the hazard categories (Table 1). This is the case for *acute toxicity* (GHS06 and GHS07), *skin corrosion/irritation* (GHS05 and GHS07), *serious eye damage/eye irritation* (GHS05 and GHS07) and *specific target organ toxicity – single exposure* (GHS07 and GHS08)*. Skin sensitisation* is the only hazard class which triggers GHS07 alone. Consequently, GHS07 was ranked as the last pictogram in the optimised testing strategies.

For the second step, the hazard classes triggering each pictogram were ordered according to the goal of each testing strategy. Testing strategy B minimises the number of tests performed. Active substances in the dataset classified for each hazard category (or group of categories) were counted for each pictogram. The most prevalent hazard classes for each pictogram were prioritised to limit the number of tests performed: once a pictogram is triggered, further testing is not necessary. Testing strategy C prioritises the use of NAMs over animal testing. All available OECD TGs were listed and attributed to each hazard class according to the BPR, the PPR and specific integrated approaches to testing and assessment when relevant. The OECD TGs relying on *in chemico*, *in vitro* and *in silico* approaches were identified (Table 2 in Supplementary Information). The exact number of animals used for each hazard class could not be quantified because of the multiple OECD TGs available for each hazard class and the unknown number of animals used for each OECD TG. The report of the European Commission on the use of animals for scientific purposes (EC, 2020) and the respective test guidelines were used to rank the hazard classes relying solely on animal experiments (such as *carcinogenicity*, *reproductive toxicity* and *specific target organ toxicity – repeated exposure*).

Testing strategy D combines testing strategies B and C to find a compromise between reducing the number of tests performed and using experimental animals.

## 3 Results and discussion

### 3.1 Hazard profiles of substances in the dataset

**Fig. 1:**
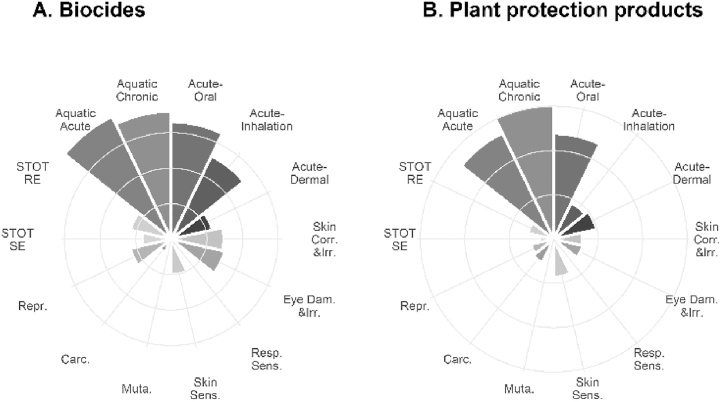
Overview of the relative number of EU-classified active substances for each hazard class in A. biocides (n = 147) and B. plant protection products (n = 1256). The exact number of active substances classified for each hazard class for biocides and plant protection products is available in Table 3 in Supplementary Information.

Fig. 1 illustrates the relative number of active substances in biocides and in plant protection products classified for each hazard class in the EU. It shows which hazards classes are most commonly assigned.

### 3.2 Content and performance of the new testing strategies

#### 3.2.1 Description of the three optimised testing strategies

As previously described, a two-step optimisation was applied. Regarding the classification of human health hazards, at the pictogram level (step 1), GHS07 is ranked last for all testing strategies in view of the precedence rules of the CLP Regulation (EC, 2019) and because GHS07 can be triggered indirectly when testing for the pictograms GHS05, GHS06 and GHS08. This avoids redundant testing, in line with strategy B. When GHS07 has not been previously triggered, testing for *skin sensitisation* can be performed. Pictograms GHS05 and GHS06 are ranked at the same level, but both are placed before pictogram GHS08 because classification for *specific target organ toxicity – single exposure* relies on human data and animal experiments from *acute toxicity* studies and *skin corrosion/irritation* studies (EC, 2019). The final ranking of the pictograms for the classification of human health hazards is 1. GHS05 or GHS06, 2. GHS08, and 3. GHS07.

The ranking of the hazard classes (step 2) differs for each testing strategy (Table 2). In line with testing strategy B, the hazard class *specific target organ toxicity – repeated exposure* ranks first for GHS08 because it is the most prevalent (Fig. 1). This means that for the 101 substances classified for this hazard class (7.2 % of the full dataset), further testing is not necessary to trigger the pictogram GHS08. With regards to testing strategy C, *aspiration toxicity*, *mutagenicity* and *specific target organ toxicity – single exposure* rank first because they rely on the physicochemical properties of the active substance (OECD, 2002), on *in vitro* studies as a first tier (OECD, 2015) and on data obtained elsewhere, respectively.

**Table 2:** Ranking of hazard classes in testing strategies B, C and D for human health (steps 1 to 13) and environmental hazards (steps 1 and 2). The underlined hazard classes rely solely on animal testing.

		Testing strategy B	Testing strategy C	Testing strategy D
Pictogram	Steps	Minimisation of number of tests	Minimisation of animals used	Compromise between B and C
GHS06: Toxic	1−3	Acute toxicity – oral	Acute toxicity – oral	Acute toxicity – oral
		Acute toxicity – dermal	Acute toxicity – dermal	Acute toxicity – dermal
		Acute toxicity - inhalation	Acute toxicity - inhalation	Acute toxicity - inhalation
GHS05: Corrosive	4	Serious eye damage/eye irritation	Skin corrosion/irritation	Skin corrosion/irritation
	5	Skin corrosion/irritation	Serious eye damage/eye irritation	Serious eye damage/eye irritation
GHS08: Health hazard	6	STOT – RE	Aspiration toxicity*	Aspiration toxicity*
	7	Reproductive toxicity	Mutagenicity	Mutagenicity
	8	Carcinogenicity	STOT – SE	STOT – SE
	9	STOT – SE	Carcinogenicity	Carcinogenicity
	10	Mutagenicity	Respiratory sensitisation*	Reproductive toxicity
	11	Respiratory sensitisation*	Reproductive toxicity	Respiratory sensitisation*
	12	Aspiration toxicity*	STOT – RE	STOT – RE
GHS07: Harmful	13	Skin sensitisation	Skin sensitisation	Skin sensitisation
GHS09: Environmental hazard	1	Aquatic - acute	Aquatic - acute	Aquatic - acute
	2	Aquatic - chronic	Aquatic - chronic	Aquatic - chronic

* No OECD test guideline available for this hazard class STOT SE: specific target organ toxicity – single exposure. STOT RE: specific target organ toxicity – repeated exposure.

Despite reducing the number of tests performed, testing strategy B does not necessarily limit the use of experimental animals because it does not take into account the method type (*in chemico*, *in vitro*, *in silico*, or *in vivo*) to evaluate each hazard class. For instance, *specific target organ toxicity – repeated exposure* is ranked first in the GHS08 group (*i.e.* all substances should be tested for this hazard class) even though it relies on animal testing. On the other hand, prioritising hazard classes for which new approach methodologies are available (testing strategy C) does not efficiently reduce the number of tests performed. In this dataset, few active substances are classified for *aspiration toxicity* and *mutagenicity*: evaluating them first does not significantly reduce the number of tests to be performed.

A compromise between the reduction of the overall number of tests performed (testing strategy B) and the number of animal tests required (testing strategy C) was also considered (testing strategy D).

Classification of environmental hazards was dealt with separately because it is independent of human health hazards. According to testing strategy B, the hazard class *aquatic toxicity - chronic* should come first because it is more prevalent than *aquatic toxicity – acute* (Fig. 1). However, in practice, chronic hazard classification can be based on acute data but not *vice versa*. For that reason, *aquatic toxicity - acute* comes first in the final testing strategies B, C and D.

#### 3.2.2 Quantitative evaluation of the optimised testing strategies

**Fig. 2:**
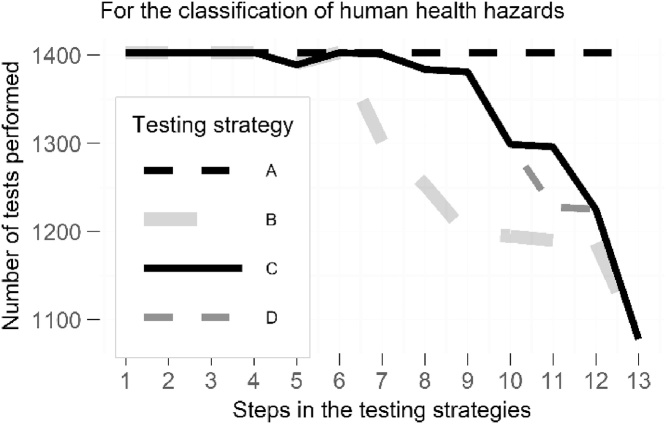
Number of tests performed for the classification of human health hazards according to testing strategies A, B, C and D. The number of tests is treated as the total number of substances (1403) in the combined dataset (biocides and plant protection products).

The number of substances tested for each hazard class and each testing strategy was counted to quantitatively evaluate and compare the three alternatives (Fig. 2). According to the current strategy A, and making the assumption that no tests were waived in the dataset, 1403 active substances are tested for each hazard class. This is not the case for the three optimised testing strategies: the higher a hazard class is ranked in each pictogram group, the fewer active substances will be tested for it. For some hazard classes such as *serious eye damage/eye irritation*, the reduction in number of tests performed was minor. Conversely, for other hazard classes like *skin sensitisation* a significant number of tests was avoided (325 tests, Table 4 in Supplementary Information). Because of the interdependence of the routes of exposure for acute toxicity, these hazard classes were not ranked. Indeed, according to BPR and PPR, while *acute toxicity – oral* should always be reported, testing for *acute toxicity – dermal* and *inhalation* depends on the exposure scenario and waivers exist. Overall, the implementation of optimised testing strategies B and D reduces the number of tests performed on the full dataset from 21,045 to 19,185 (in the case of B) and to 19,778 (in the case of D), representing a decrease of 8.8 % and 6.0 % respectively (1860 and 1267 fewer tests) (Table 4 in Supplementary Information).

Since testing strategies B, C and D were built with the boundary condition that they must indicate the same levels of concern, the distribution and the frequency of the pictograms triggered across the dataset remain the same.

## 4 Conclusions and perspectives

Current approaches to regulatory testing are generally based on standard checklists of information requirements which underpin both hazard-based and risk-based approaches to risk management. In the case of biocides and plant protection products, most of the tests conducted for the purpose of hazard classification also support risk assessment. In this paper, we focus specifically on the hazard-based approach. While the classification of a chemical triggers risk management measures, the relationship between hazard classification and risk management is not a one-to-one relationship. This has the consequence that many tests will have no impact on the subsequent risk management. In other words, more information on a chemical does not necessarily result in more protection. Therefore, testing strategies can be optimised by understanding when additional data do not influence the measures for chemical safety. The idea of waiving specific toxicity studies on a case-by-case basis is not uncommon in regulatory practice but could be made more systematic and efficient.

This study defines and evaluates three optimised testing strategies for improving the efficiency of classification and labelling while indicating the same levels of concern in a given dataset. Testing strategy B reduces the number of tests performed by 8.8 % (1860 tests). Testing strategy C prioritises the use of NAMs, thereby saving animals. Testing strategy D is a compromise between B and C (reduction in number of tests by 6.0 %).

The proposed ranking of the pictograms in the optimised testing strategies is based on precedence rules and depends solely on the conditions set in the CLP Regulation (EC, 2019). Similarly, the order of the hazard classes in testing strategy C depends on the method type and is independent of the dataset. In contrast, the order of the hazard classes in testing strategies B and D originates from the distribution of hazard classes in the dataset (Fig. 1).

Other criteria than the overall number of tests performed and the use of experimental animals could have been chosen to develop the optimised testing strategies. For example, the cost of the assays performed for each hazard class, their duration, and complexity or criteria related to the animals such as the severity of the tests required or the species involved could have been selected. A rigorous estimation of the number of animals used for toxicity testing of chemicals would be a challenge, subject to several uncertainties. Considerably more work would therefore be needed to provide quantitative evidence of the animal reduction benefit of new approach methodologies and optimised testing strategies.

The testing strategies presented here could be further optimised. According to the chemical similarity hypothesis, compounds of similar structure have a similar mode of action, thereby leading to a similar hazard *in vivo*. Based on this hypothesis, quantitative structure-activity relationship (QSAR) models and read-across are applied in a regulatory context to predict physical or chemical properties of compounds and their specific hazards (Gocht et al., 2015). For this reason, the integration of physicochemical properties as a first tier, prior to performing testing for the first hazard class, would enable the classification for some hazard classes to be predicted, thereby minimising testing even more. Similarly, mapping the chemical structure and physicochemical properties of substances included in a product type category could be of interest to investigate whether the toxicity profile could be predicted based on such information rather than toxicity testing.

Integrating data across hazard classes is another way of further optimising the testing strategies: do some tests performed for one hazard class provide information for another hazard class? Mapping all the observations made to classify a compound would help identify intersections between hazard classes to inform specific targeted tests rather than standard test sets. Alternatively, endpoint-specific integrated testing strategies can be developed. As outlined in the EU-funded OSIRIS project (2007–2011), such strategies have proved useful to gain efficiencies for regulatory decision making on chemicals by taking advantage of existing information. Data gaps can be identified after the gathering and evaluation of all existing information and the relevant data necessary to fill the gaps can be obtained while prioritising non-testing and *in vitro* methods over animal testing (Vermeire, 2013). Similarly, our approach is designed to avoid unnecessary experiments, and contributes to reducing animal testing for classification and labelling while indicating the same levels of concern as the basis for risk management measures aimed at protecting human health and the environment.

Optimised testing strategies can also be used to identify where the development and the implementation of NAMs would have most impact. The availability of non-animal tests for early hazard classes will significantly reduce the use of experimental animals, and will potentially make regulatory testing more time- and cost-efficient.

This proof-of-concept study is intentionally based on a simplistic approach using pictograms as a starting point for optimising testing strategies. The GHS labelling system is hazard-based, and as such the same pictogram can group together endpoints with different exposure scenarios. For example, *specific target organ toxicity – single exposure*, resulting from a single exposure to a chemical, and *carcinogenicity*, related to exposure at lower doses but over a longer period, trigger the same pictogram GHS08. In reality, pictograms are not the only descriptors reflecting the level of concern for the population and the environment. However, the intention here is not to propose a re-design of the GHS, but rather to emphasise that additional information on chemical properties does not necessarily translate into a higher level of protection. This means that efficiencies can be gained in the testing needed to indicate a given level of concern, at least within a hazard-based risk management approach. We did not investigate how efficiencies could be gained in the risk-based approach, but other studies have shown that not all of the toxicity tests routinely conducted are actually used to derive the point of departure for risk assessment (Paul Friedman, 2019; Beames et al., 2020).

The dataset used in this study can be considered robust because data are required for most toxicity endpoints and are evaluated by expert panels for the harmonised authorisation of active substances in biocides and plant protection products. However, due to the severe restrictions on active substances with carcinogenic, mutagenic, and reprotoxic properties, the dataset used in this study contains few CMRs.

Although the optimised testing strategies developed here are based on specific datasets, it is also recognised that no single testing strategy is applicable to all contexts of use. Important considerations for defining testing strategies include, for example, the exposure scenario of interest, the need to address specific health or environmental concerns, the availability of hazard information, regulatory options for generating new data, as well as economic and animal welfare considerations. Nevertheless, the concept of optimised testing strategies has the potential to be applied to other chemical groups, including chemicals regulated under REACH to support waiving of additional testing to fulfil data requirements. Even though this work was based on data from European sources, testing strategies could be elaborated similarly in jurisdictions of any country based on the GHS. In general, there is a need to improve efficiencies in the assessment process, irrespective of the decision-making context. This work is thus intended as a proof-of-concept and thought-starter for developing more efficient, but equally effective, safety assessment approaches.

## Declaration

The scientific output expressed does not imply a policy position of the European Commission.

## Declaration of Competing Interest

The authors report no declarations of interest.
